# The Influence of Reproductive Experience on Milk Energy Output and Lactation Performance in the Grey Seal (*Halichoerus grypus*)

**DOI:** 10.1371/journal.pone.0019487

**Published:** 2011-05-11

**Authors:** Shelley L. C. Lang, Sara J. Iverson, W. Don Bowen

**Affiliations:** 1 Department of Biology, Dalhousie University, Halifax, Nova Scotia, Canada; 2 Population Ecology Division, Bedford Institute of Oceanography, Dartmouth, Nova Scotia, Canada; University of Pretoria, South Africa

## Abstract

Although evidence from domestic and laboratory species suggests that reproductive experience plays a critical role in the development of aspects of lactation performance, whether reproductive experience may have a significant influence on milk energy transfer to neonates in wild populations has not been directly investigated. We compared maternal energy expenditures and pup growth and energy deposition over the course of lactation between primiparous and fully-grown, multiparous grey seal (*Halichoerus grypus*) females to test whether reproductive experience has a significant influence on lactation performance. Although there was no difference between primiparous females in milk composition and, thus, milk energy content at either early or peak lactation primiparous females had a significantly lower daily milk energy output than multiparous females indicating a reduced physiological capacity for milk secretion.

Primiparous females appeared to effectively compensate for lower rates of milk production through an increased nursing effort and, thus, achieved the same relative rate of milk energy transfer to pups as multiparous females. There was no difference between primiparous and multiparous females in the proportion of initial body energy stores mobilised to support the costs of lactation. Although primiparous females allocated a greater proportion of energy stores to maternal maintenance versus milk production than multiparous females, the difference was not sufficient to result in significant differences in the efficiency of energy transfer to pups. Thus, despite a lower physiological capacity for milk production, primiparous females weaned pups of the same relative size and condition as multiparous females without expending proportionally more energy. Although reproductive experience does not significantly affect the overall lactation performance of grey seals, our results suggest that increases in mammary gland capacity with reproductive experience may play a significant role in the age-related increases in neonatal growth rates and weaning masses observed in other free-ranging mammals.

## Introduction

Changes in reproductive success with age have been observed in many species of birds and mammals [1], [2]. The general pattern is that reproductive performance initially increases with age, reaches a plateau and then either remains constant or shows a progressive decline into old age. Because these age-specific changes play an important role in our understanding of the evolution of life history patterns, behavioural strategies and population dynamics, identifying the factors which underlie these patterns is of considerable interest. One hypothesis put forward to explain the initial increase in the reproductive performance of individuals is that improvements occur, in part, through increased reproductive experience, either as a function of changes in the behaviours associated with reproduction and/or as a result of physiological development and maturation [3], [4]. Although this hypothesis has received considerable attention in the study of the patterns of early reproductive performance in free-ranging birds (e.g. [5], [6]), relatively little attention has been paid to how cycles of pregnancy, parturition and lactation may influence aspects of reproductive performance in young female mammals.

In mammals, the most significant determinant of offspring growth during lactation is the ability of females to transfer milk energy to their neonates. Lactation is the most energetically demanding period in the life of a female mammal [7] and females offset these demands by increasing energy intake and/or mobilizing body energy stores. Because females of many species typically begin to reproduce before reaching full adult body size, lower neonatal growth rates and weaning masses in first time breeders are often assumed to result from limits on the energy available for milk production as a consequence of smaller absolute levels of body energy stores and/or a smaller gut capacity, combined with the need to partition resources between maternal growth and supplying nutrients to offspring. However, while increases in body mass with age in young females often correlate with improved lactation performance (e.g. [8]–[11]), energy availability may not be the only factor. Studies in both domestic and laboratory species demonstrate that the mammary gland is not fully mature at first parturition and that repeated cycles of pregnancy, parturition and lactation play a critical role in the progressive development of mammary gland capacity [12]–[15]. Thus, primiparous females may have significantly lower rates of milk production compared to multiparous females regardless of their levels of body energy stores and/or energy intake [14], [16], [17]. Primiparous females may also exhibit lower levels of maternal care or inefficient maternal behaviours compared to multiparous females resulting in a reduced delivery of milk energy to offspring. Studies in both laboratory and domestic species demonstrate that cycles of pregnancy, parturition and lactation play a direct role in the development of maternal behaviours through a series of complex neurological changes which are initiated during a female's first reproductive effort by the hormones associated with pregnancy, parturition and the onset of lactation. These neurological changes are reinforced by the subsequent interactions with offspring and are then carried over into the next reproductive cycle. As a result, in some species, multiparous females show significant improvements in both the intensity and quality of maternal behaviours compared to primiparous females (see [18], [19] for reviews). Nevertheless, comparison of the level of milk energy transfer to neonates between first time breeders and multiparous females in natural populations has yet to be directly investigated.

The factors influencing the transfer of milk energy to offspring in fully-grown multiparous females have been well studied in grey seals (*Halichoerus grypus*). Like most other large bodied phocid seals (Family Phocidae), grey seal females are capital breeders, relying entirely on the body energy reserves acquired prior to parturition to support both the energetic costs of milk production and their own metabolic overhead [20]. Females give birth to a single pup, there is no alloparental or paternal support and pups consume only milk during the lactation period. Females remain ashore with their pup throughout the relatively brief lactation period (16–18 d, [21]) during which females secrete large quantities (average 3.2 kg d^−1^) of high energy milk (40–60% fat, [20], [22], [23]). At the end of lactation females abruptly wean their pups and depart the breeding colony. Pups must then rely on the energy stores acquired during the lactation period to survive a 3–4 week post-weaning fast and the transition to nutritional independence [24]. In this species, both the body mass and condition of pups at weaning have a significant influence on post-weaning survival with larger, fatter pups having a greater probability of survival to 1 yr of age [25].

Because grey seal females begin to reproduce at 4–6 years of age, well before reaching full adult body size in their early to middle teens [8], the reduced lactation performance of primiparous females (measured as pup weaning mass) has been attributed to their smaller absolute levels of body energy stores alone [8]. However, behavioural observations indicate that primiparous grey seal females may face additional constraints on milk energy transfer. Compared to multiparous females, primiparous grey seal females have a significantly higher frequency of nursing bouts, suggesting that they may have a lower physiological capacity for milk secretion [26]. In addition, primiparous females exhibit a significantly higher level of activity throughout lactation compared to multiparous females, suggesting that they may allocate a greater proportion of their available body energy stores to maternal maintenance versus milk production and, thus, pup growth [26].

We compared maternal energy expenditures and pup growth and energy deposition over the course of lactation between primiparous and fully-grown, multiparous grey seal females to test whether reproductive experience has a significant influence on lactation performance. Because the development of both the mammary gland and maternal behaviours are affected by the hormones associated with pregnancy and parturition in addition to those associated with lactation [18], [19], [27], [28], reproductive experience is defined here as including any previous observation of pregnancy regardless of whether females subsequently nursed a pup. We tested two hypotheses. The first was that the lactation performance of primiparous females is reduced compared to multiparous females as a result of a lower physiological capacity to deliver milk energy. The second hypothesis was that primiparous females allocate a greater fraction of available energy to maternal maintenance versus milk production.

## Materials and Methods

### Field Procedures

The study was conducted on Sable Island (43°55′N, 60°00′W), located approximately 300 km ESE of Halifax, Nova Scotia, Canada during the 2003 through 2005 breeding seasons (late December through to early February). Females in this population begin reproducing at 4–6 years of age and can continue to reproduce to age 30 or more [8]. Study females were a subset of those which were permanently marked as pups between 1973 and 1989 and between 1998 and 2002 with unique, hot-iron brands shortly after weaning and, thus, were of known age. As with other grey seal colonies [29], [30], the Sable Island grey seals exhibit a strong philopatry, with an estimated fidelity rate of 98.4% (W. D. Bowen, unpublished data).Weekly whole island censuses of all branded individuals combined with daily surveys throughout the colony during the breeding season have been conducted in this population since 1983 (see [8] for details) and, thus, the reproductive histories of all females in the study were known. The 15 multiparous females in the study were between 14 and 31 years of age and had each been observed pregnant and/or rearing a pup in a minimum of 7 previous breeding seasons. The 17 primiparous females were 5 years of age. Females were considered primiparous if they had not been observed pregnant or rearing a pup in a previous breeding season. Given the frequency of whole island censuses and colony surveys, it is highly unlikely that a female returning to the colony to give birth would not have been detected. Non-pregnant females are rarely observed among lactating females on Sable Island. Of the 311 females from the 1998–2002 cohorts which recruited to Sable Island, none were sighted on Sable Island or at any other colony in breeding seasons prior to the first year they were observed with a pup. Therefore, we are confident that the first year a female was observed with a pup, she was primiparous. All females used in this study had known parturition dates.

On day 3 postpartum (early lactation) adult females were captured using a hinged pole net. Females and pups were weighed to the nearest 0.5 kg and then given isotopically labelled water to determine total body water, water flux and milk intake according to Iverson et al. [20] and Mellish et al. [22]. Day 3 postpartum was selected for initial sampling to allow the pair bond to develop between females and their pups and, thereby, minimize the risk of abandonment as a result of handling and to permit direct comparison with previous studies in this population. Females were given an intramuscular injection of a precisely weighed dose (approximately 5 g @ 0.5 mCi ml^−1^) of tritiated water (^3^H_2_O; MP Biomedicals, OH, USA). Pups were given a precisely weighed dose (approximately 3 g kg^−1^) of 99.9% deuterium oxide (D_2_O; Cambridge Isotope Laboratories, Inc., MA, USA) by gastric intubation. Blood samples (10 ml) were taken from both females and pups at 2.0 and 2.5 h post administration via the extradural vein to determine the concentration of labelled water and to confirm that equilibration had occurred. Blood samples were centrifuged for 20–30 minutes and serum aliquots stored frozen at −20°C until analysis.

Following the final blood sampling of both the female and pup, females were mildly sedated with an intravenous injection of diazepam (5.0 ml @ 5 mg ml^−1^, Sandoz Canada Inc., QC, Canada) and given an intramuscular injection of oxytocin (1.5 ml @ 20 IU ml^−1^, Vétoquinol Canada, QC, Canada) to facilitate milk letdown. 60 ml of milk was then collected by suction and stored in 30 ml Nalgene bottles at −20°C until analysis. Prior to release pups were given an individually numbered hind-flipper tag (Rototag, Dalton ID Systems Limited, Oxon, UK) to permit post-weaning identification.

Pairs were resampled between days 10 and 13 postpartum (peak lactation). Based on an average lactation length of approximately 15 days for 5 year old grey seal females [8], day 10 postpartum was initially selected as the second sampling point to provide a high probability of obtaining data from all pairs prior to weaning. However, following an observed average lactation length of 17 days (range 15–20 days) in the primiparous females studied in 2003 (n = 5), sampling was targeted for day 12 postpartum for the subsequent seasons in order to cover a greater proportion of the lactation period. Severe weather conditions and the demands of concurrent sampling resulted in some peak lactation samples being taken at day 11 postpartum (n = 2) or day 13 postpartum (n = 2). Following capture and weighing, a single blood sample (10 ml) was taken from both the female and pup to determine the concentration of labelled water. Isotopes were then readministered and blood and milk samples collected as described above with the exception that D_2_O was given to pups at a dose of approximately 1 g kg^−1^. Pairs were sighted daily throughout the lactation period to ensure that the female and pup were still together and to obtain an accurate date of weaning. On the day of weaning, which is marked by the departure of the female from the colony, pups were weighed to the nearest 0.5 kg.

All sampling protocols were conducted in accordance with the requirements of the Canadian Council on Animal Care and were approved by Dalhousie University's Committee on Laboratory Animals (protocol numbers 01-087, 03-095 and 05-115).

### Sample Analyses

Milk samples were analysed for protein (macro-Kjeldahl [31], fat (Roese-Gottlieb [32] and dry matter content (as described in Iverson et al. [20]). Milk samples were not analysed for carbohydrate content as it has been previously demonstrated that this is a very minor component of phocid milks [33], as was confirmed by the low residuals from the sum of fat and protein compared to dry matter.

Female serum samples were distilled in triplicate (50 µl aliquots) using the method of Ortiz et al. [34]. The ^3^H activity of the distilled sera and of the injectant were determined using a Beckman LS 6000TA liquid scintillation counter (Beckman Coulter, Inc., CA, USA). Values for triplicates were averaged. Pup serum samples were distilled using the method of Oftedal and Iverson [35]. The D_2_O concentration of the distilled sera was measured using a single-beam Fourier transform, infrared spectrophotometer (Perkin-Elmer FT-IR Paragon 1000; Perkin-Elmer, MA, USA), using gravimetrically prepared standards and distilled water as a reference. All distilled sera samples were analysed for D_2_O concentration in triplicate and the values averaged.

### Calculations and Data Analyses

The isotope dilution space of females and pups was calculated for early and peak lactation following Iverson et al. [20]. Total body water (kg) was estimated from the regression equation for isotope dilution space on total body water derived for pinnipeds by Bowen and Iverson [36]. Total body fat (%) and protein (%) were estimated from total body water using the equations developed for grey seals by Reilly and Fedak [37].

The total daily water intake (TWI, kg d^−1^) and daily milk intake (MI, kg d^−1^) of pups between early and peak lactation were calculated as described in Oftedal and Iverson [35]. MI was estimated using the following equation:
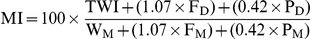
where F_D_ and P_D_ are the daily fat and protein deposition rates of the pup (kg d^−1^), respectively, between early and peak lactation and W_M_, F_M_ and P_M_ are the average values for the percent water, fat and protein content of the milk, respectively. In grey seals, milk dry matter and fat content increase from parturition until reaching peak values at approximately day 8 postpartum and then remain relatively stable over the remainder of lactation [20]. To account for the non-linear changes in both milk water and fat content over the measurement period, we calculated weighted harmonic means for W_M_ and F_M_ following Lang et al. [23]. After an initial decline shortly after parturition, milk protein content remains relatively constant throughout the remainder of lactation in grey seals [20] and, therefore, an average of the early and peak lactation values was used for P_M_. Gross energy contents of tissues and milk and daily rates of energy loss (females) or gain (pups) were calculated using the values of 39.3 MJ kg^−1^ and 23.6 MJ kg^−1^ for fat and protein, respectively [38].

Two primiparous females abandoned their pups at day 4 postpartum and, thus, data for these pairs are only included in the early lactation comparisons for milk composition and body mass and condition. Data for these pairs were not included in the analyses of lactation length or weaning mass. Data for all remaining pairs at both early and peak lactation were used in the analyses with the exception of one pup of a primiparous female. In this one case, the estimated daily milk energy intake of the pup was consistent with that estimated for the pups of other primiparous females, however, the pup's energy storage efficiency was an extreme outlier (>3 SD from the overall mean of 70.8±0.75%, n = 29) with the pup storing less than half (44.2%) of its estimated milk energy intake. Thus, only the data on milk intake and milk energy intake for this pup were included in the analyses. For individuals sampled at days 10, 11 or 13 postpartum, values for mass and body composition were adjusted to day 12 postpartum prior to analysis using the rate of change per day for each component for that individual. Values for maternal mass and body energy stores at parturition were estimated using the rate of change per day for each component for that female. All mass-specific values were calculated relative to maternal parturition mass. Percentage data were arcsine transformed prior to analysis. Statistical comparisons between groups were made using the t-test for independent samples. All statistical analyses were conducted in SPSS version 11.0 (SPSS Inc., IL, USA). Standard errors are reported throughout.

## Results

**Figure 1:**
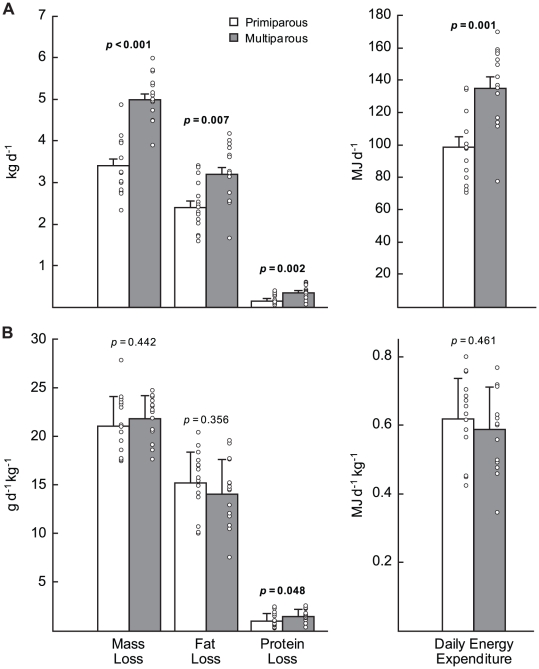
Daily energy expenditures of primiparous and multiparous grey seal females during lactation. Absolute (A) and mass specific (B) daily mass, fat and protein loss and daily energy expenditure of primiparous (n = 15) and multiparous (n = 15) grey seal females between early and peak lactation. Values are means ± standard error, open circles are raw values.

Although primiparous females weighed less than multiparous females at day 3 postpartum, there was no significant difference in body composition between the groups (Table 1). Between early and peak lactation, primiparous females lost less body mass per day, both as fat and protein and, therefore, had significantly lower absolute rates of daily energy expenditure than did multiparous females (Fig. 1A). Primiparous females had a significantly lower mass-specific rate of protein loss than multiparous females, but there was no significant difference in mass-specific fat loss per day or in mass-specific mass loss per day between the groups (Fig. 1B). Because fat loss accounted for a greater proportion of daily energy expenditure than protein (94.8% versus 5.2%), there was no significant difference in mass-specific daily energy expenditure between the groups (Fig. 1B).

**Table 1:** Body mass and composition of primiparous and multiparous grey seal females at day 3 and day 12 postpartum.

	Reproductive Status				
	Primiparous		Multiparous		*p*
**Day 3 Postpartum**	**n = 17**		**n = 15**		
Mass (kg)	150.1±3.02	(128.0–176.0)	216.6±4.74	(190.0–256.0)	<**0.001**
Water (%)	048.4±0.71	(43.0–53.2)	050.6±0.73	(43.0–56.1)	0.123
Fat (%)	033.9±1.05	(25.4–41.9)	030.7±1.08	(22.6–38.5)	0.126
Protein (%)	015.6±0.30	(13.3–18.0)	016.5±0.31	(14.3–18.8)	0.120
**Day 12 Postpartum**	**n = 15**		**n = 15**		
Mass (kg)	119.0±2.00	(106.5–138.5)	171.6±4.48	(147.1–211.5)	<**0.001**
Water (%)	055.2±1.07	(48.5–61.7)	056.7±1.16	(48.7–63.7)	0.333
Fat (%)	023.9±1.57	(14.4–33.8)	021.7±1.71	(11.5–33.8)	0.319
Protein (%)	018.4±0.45	(15.6–21.1)	019.1±0.49	(15.7–22.0)	0.337

Values are means ± standard errors, ranges are in parentheses.

**Figure 2:**
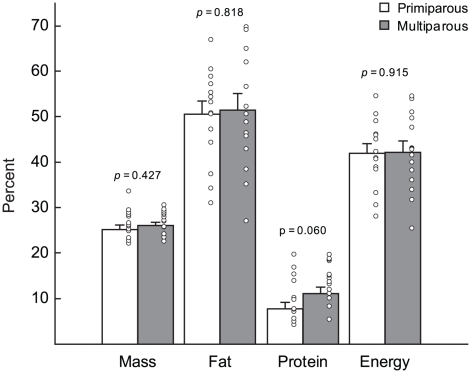
Proportion of energy stores mobilized by primiparous and multiparous females during lactation. Proportion of initial mass, fat, protein and energy stores mobilized by primiparous (n = 15) and multiparous (n = 15) females between parturition and day 12 postpartum. Values are means ± standard error, open circles are raw values.

Multiparous females lost significantly greater absolute amounts of energy stores between early and peak lactation than primiparous females. However, there were no significant differences between the groups in the proportions of initial mass, fat, protein or energy stores mobilized to support the costs of lactation between parturition and day 12 postpartum (Fig. 2). Thus, there were no significant differences in body composition between primiparous and multiparous females at day 12 postpartum (Table 1).

**Figure 3:**
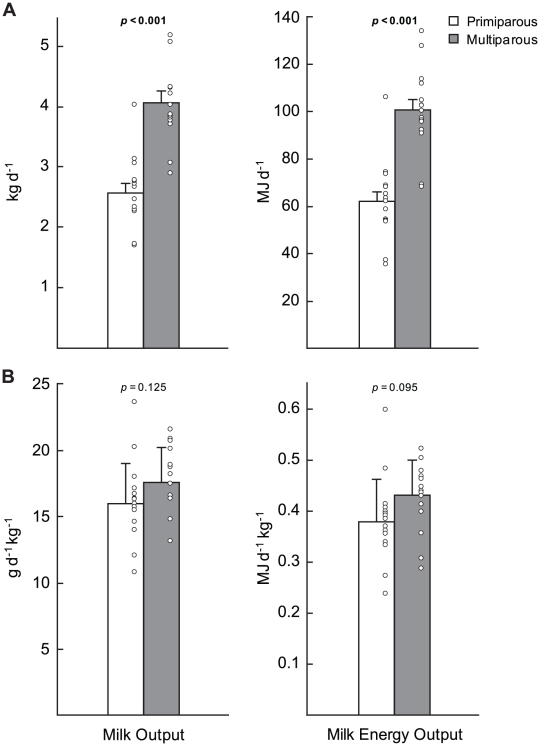
Daily milk output and daily milk energy output of primiparous and multiparous grey seal females. Absolute (A) and mass specific (B) daily milk output and daily milk energy output of primiparous (n = 15) and multiparous (n = 15) grey seal females between early and peak lactation. Values are means ± standard error, open circles are raw values.

**Figure 4:**
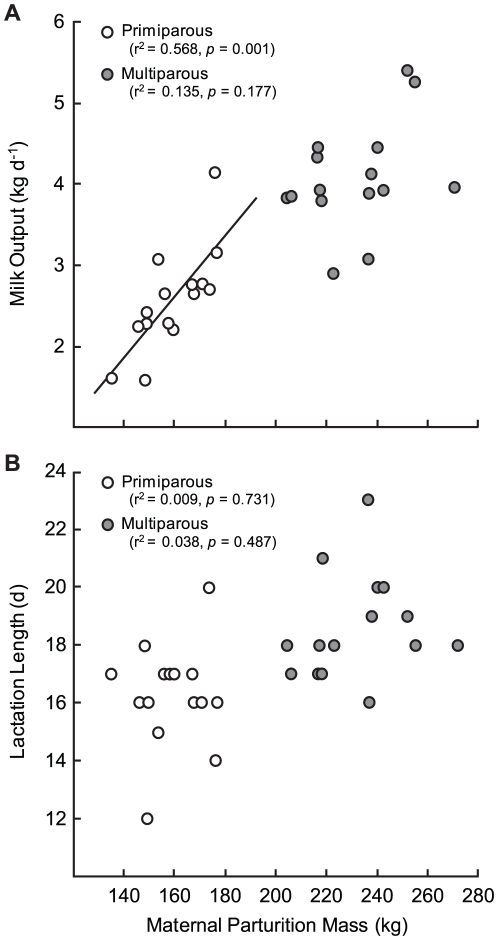
Relationship between maternal parturition mass and daily milk output and lactation length. Relationship between maternal parturition mass and daily milk output (A) and lactation length (B) in primiparous (n = 15) and multiparous (n = 15) grey seal females.

Milk composition and, thus, milk energy content at early and peak lactation did not differ significantly between primiparous and multiparous females (Table 2). Primiparous females had significantly lower average rates of daily milk output and, therefore, daily milk energy output than did multiparous females between early and peak lactation (Fig. 3A). However, there were no significant differences between the groups in mass-specific rates of milk output or milk energy output (Fig. 3B). There was no relationship between maternal parturition mass and daily milk output in multiparous females, but daily milk output was positively related to maternal parturition mass in primiparous females (Fig. 4A). The proportion of daily energy expenditure devoted to milk energy output between early and peak lactation was significantly less for primiparous females (63.1±3.8%, range 42.3–88.2%) compared to multiparous females (75.0±3.22%, range 57.1–94.3%; *p* = 0.024).

**Table 2:** Proximate composition of the milk of primiparous and multiparous grey seal females at early (day 3 postpartum) and peak (day 10–13 postpartum) lactation.

	Reproductive Status				
	Primiparous		Multiparous		*p*
**Early Lactation**	**n = 17**		**n = 15**		
Water (%)	41.7±0.79	(36.4–47.6)	41.0±1.03	(32.2–48.0)	0.569
Dry Matter (%)	58.3±0.79	(52.4–63.6)	59.0±1.03	(52.0–67.8)	0.569
Protein (%)	08.7±0.15	(7.8–10.0)	08.8±0.22	(7.8–10.3)	0.657
Fat (%)	46.7±0.80	(39.9–51.0)	47.3±0.90	(41.1–53.7)	0.593
Energy (MJ kg^−1^)	20.4±0.32	(24.3–26.8)	20.6±0.38	(23.9–28.0)	0.577
**Peak Lactation**	**n = 15**		**n = 15**		
Water (%)	28.3±0.48	(25.2–31.6)	27.1±0.75	(23.2–32.8)	0.180
Dry Matter (%)	71.7±0.48	(68.4–74.8)	72.9±0.75	(67.2–76.8)	0.180
Protein (%)	08.8±0.13	(8.1–9.6)	09.1±0.20	(8.2–10.8)	0.394
Fat (%)	59.8±0.55	(56.4–63.5)	61.2±0.86	(55.8–66.2)	0.183
Energy (MJ kg^−1^)	25.6±0.20	(24.3–26.8)	26.1±0.32	(23.9–28.0)	0.128

Values are means ± standard errors, ranges are in parentheses.

Pups of primiparous females were lighter than those of multiparous females at day 3 postpartum (Table 3). However, pup mass at day 3 postpartum as a proportion of maternal parturition mass was not significantly different between primiparous and multiparous females and there were no significant differences in pup body composition between the groups (Table 3). Pups of primiparous females gained significantly less mass, fat, protein, and energy per day compared to pups of multiparous females (Table 4). However, there were no significant differences in the energy storage efficiency of pups (100× pup daily energy gain/daily milk energy intake) or in the overall efficiency of energy transfer to pups (100× pup daily energy gain/maternal daily energy expenditure) between the groups (Table 4). Pups of primiparous females weighed significantly less than pups of multiparous females at day 12 postpartum (Table 3). However, as a proportion of maternal parturition mass, pup mass at day 12 postpartum did not differ significantly between primiparous and multiparous females and there were no significant differences in pup body composition between the groups (Table 3). As a proportion of weaning mass, pup mass at day 12 postpartum also did not differ between primiparous (91.9±1.81%) and multiparous females (86.5±2.25%; *p* = 0.084).

**Table 3:** Body mass and composition of pups of primiparous and multiparous grey seal females at days 3 and 12 postpartum and at weaning.

	Reproductive Status								
	Primiparous			Multiparous				*p*	
**Day 3 Postpartum**	**n = 16**		**n** = **15**						
Mass (kg)	16.7±0.74	(11.0–24.5)		23.3±0.69		(16.0–29.5)		**<0.001**	
Mass (% of MPM*a*)	10.6±0.38*b*	(8.2–13.9)		10.1±0.42		(6.8–13.6)		0.394	
Water (%)	65.1±0.74	(60.0–69.9)		64.0±0.69		(60.0–69.9)		0.287	
Fat (%)	9.4±1.09	(2.4–16.9)		11.1±1.01		(2.3–16.8)		0.306	
Protein (%)	22.6±0.31	(20.4–24.6)		22.1±0.29		(20.5–24.6)		0.286	
**Day 12 Postpartum**	**n = 14**			**n** = **15**					
Mass (kg)	32.0±1.80	(19.0–49.5)		47.6±1.65		(31.5–55.0)		**<0.001**	
Mass (% of MPM)	19.9±0.85	(14.3–28.1)		20.6±0.80		(13.3–25.3)		0.539	
Water (%)	47.3±0.58	(43.5–50.6)		46.6±0.79		(40.8–54.1)		0.500	
Fat (%)	35.5±0.85	(30.6–41.2)		36.5±1.16		(25.5–45.1)		0.519	
Protein (%)	15.1±0.24	(13.5–16.5)		14.8±0.33		(12.4–18.0)		0.488	
**Weaning** *c*	**n = 14**			**n = 15**					
Mass (kg)	34.6±1.62	(24.0–50.5)		55.0±1.39		(47.0–67.0)		**<0.001**	
Mass (% of MPM)	21.6±0.72	(17.8–28.7)		23.9±0.73		(19.0–28.8)		0.242	
Water (%)	45.7±0.53	(42.4–50.4)		44.3±0.86		(38.2–50.1)		0.186	
Fat (%)	38.0±0.79	(33.4–42.7)		40.0±1.27		(31.4–48.9)		0.192	
Protein (%)	14.4±0.23	(13.1–15.7)		13.8±0.36		(11.3–16.3)		0.181	

a maternal parturition mass.

b n = 14 (see text for details).

c body composition at weaning estimated using pup mass gain per day from day 12 postpartum to weaning and assuming that the proportion of water, fat and protein gained per kg remained constant.

Values are means ± standard errors, ranges are in parentheses.

**Table 4:** Daily energy intake and deposition of pups of primiparous and multiparous grey seal females between early and peak lactation.

	Reproductive Status				
	Primiparous		Multiparous		*p*
	**n = 14**		**n = 15**		
Milk energy intake (MJ d^−1^)	61.7±4.30*a*	(35.5–105.6)	100.2±4.60	(68.3–133.7)	**<0.001**
Mass gain (kg d^−1^)	1.7±0.11	(0.9–2.8)	2.7±0.11	(1.7–3.3)	**<0.001**
Fat gain (kg d^−1^)	1.1±0.08	(0.6–1.8)	1.7±0.08	(1.0–2.3)	**<0.001**
Protein gain (kg d^−1^)	0.11±0.010	(0.06–0.19)	0.21±0.017	(0.08–0.34)	**<0.001**
Energy stored (MJ d^−1^)	45.6±3.21	(24.4–75.8)	70.4±3.18	(44.6–94.1)	**<0.001**
Storage efficiency (%)*b*	71.4±0.97	(65.6–77.3)	70.4±1.14	(61.4–75.7)	0.517
Transfer efficiency (%)*c*	46.3±3.03	(30.9–65.1)	52.5±7.35	(40.7–63.0)	0.092

a n = 15 (see text for details).

b 100× (daily energy intake/daily energy gain).

c 100× (pup daily energy gain/maternal daily energy expenditure).

Values are means ± standard errors, ranges are in parentheses.

Despite large variability, the average duration of lactation was significantly shorter in primiparous females (16.4±0.39 d) compared to multiparous females (18.6±0.47 d; *p*<0.001; Fig. 4B). There was no relationship between maternal parturition mass and lactation length for either primiparous or multiparous females (Fig. 4B).

Average pup mass gain per day between day 12 postpartum and weaning declined significantly in pups of both primiparous (from 1.7±0.11 to 0.6±0.12 kg d^−1^, *p*<0.001, n = 13, paired t-test) and multiparous females (from 2.7±0.11 to 1.1±0.16 kg d^−1^, *p*<0.001, n = 15, paired t-test) and declined by similar percentages (primiparous, 58.7±9.60%; multiparous, 57.8±6.95%; *p* = 0.939). Assuming that the proportions of water, fat and protein gained per kg for each pup remained constant until weaning, there was no significant difference in the estimated body composition of pups at weaning between the groups (Table 3). There was no relationship between weaning mass and body composition of pups of either primiparous (r^2^ = 0.230, *p* = 0.083) or multiparous females (r^2^ = 0.004, *p* = 0.830). Consistent with the relationship between daily milk output (Fig. 4A) and maternal parturition mass, pup weaning mass was positively related to maternal postpartum mass in primiparous females (r^2^ = 0.568, *p* = 0.001) but not in multiparous females (r^2^ = 0.135, *p* = 0.177).

We tested whether multiparous females invested a greater proportion of their initial energy stores by the time of weaning as a consequence of their longer lactation length. To do this, we estimated the energy expended by females between peak lactation and weaning and added this to the estimated energy expenditure between parturition and peak lactation. For each female her daily energy expenditure between peak lactation and weaning was calculated by estimating her daily milk energy output (MJ d^−1^) from her pup's mass gain over this period from the regression of pup mass gain (PMG, kg d^−1^) on milk energy intake (MEI) between early and peak lactation (where PMG = MEI*0.027–0.011, r^2^ = 0.952, n = 29; see Figure S1) and assuming that the energy a female devoted to maternal maintenance per day (the difference between daily energy expenditure and daily milk energy output) between early and peak lactation remained the same between peak lactation and weaning. The estimated proportion of initial energy stores mobilized to support the costs of lactation did not differ (*p* = 0.186) between primiparous (50.0±2.7%, n = 14, range 32.0–62.2%) and multiparous females (54.4±2.5%, n = 15, range 35.0–69.0%).

## Discussion

Our study appears to be the first in a wild mammal to directly compare the level and efficiency of milk energy transfer to offspring between primiparous and multiparous females. We found that first time breeders performed as well as fully-grown multiparous females. In contrast to laboratory species [39], [40], primiparous grey seal females had the same energy transfer efficiency as multiparous females and were capable of weaning pups of the same relative mass and condition as multiparous females without investing proportionally more energy. Thus, there appears to be no trade-off in these young, still growing females between allocation of energy to offspring and maternal requirements. Our results also suggest that laboratory species may not be good models for studying some aspects of lactation performance in wild species.

Milk composition and thus, milk energy content, did not differ between primiparous and multiparous females at either early or peak lactation (Table 2) demonstrating that changes in milk composition followed the same pattern in both groups over lactation. These results are consistent with those from both domestic and laboratory species (e.g. [16], [41]) and suggest that repeated cycles of pregnancy, parturition and lactation do not affect nutrient partitioning by the mammary gland in grey seals. The variation in milk composition observed among primiparous females in the present study was similar to that observed for fully-grown, multiparous females ([23], this study). This, coupled with the fact that milk composition is not influenced by variation in body size, levels of body energy stores or female age in grey seals [22], [23] suggests that the proximate composition and, thus, energy content of the milk of individual females is relatively constant throughout their reproductive life.

Previous work demonstrated that primiparous females spent significantly more time nursing their pups than multiparous females [26]. Despite this greater nursing effort, the average daily milk output (and, thus, daily milk energy output) was significantly lower for primiparous females compared to multiparous females (Fig. 3A). Daily milk output is primarily determined by the number of secretory cells in the mammary gland [42], [43]. This suggests that, like domestic and laboratory species [14], [15], the mammary glands of primiparous grey seal females have a lower volume of secretory tissue at first parturition and, thus, a reduced capacity for milk secretion compared to multiparous females. Although offspring size positively influences milk production in some domestic species (e.g. [44]), there is no relationship between a pup's birth mass and subsequent daily milk intake in grey seals (see Text S1, Figure S2). Thus, it is unlikely that the reduced daily milk output of primiparous females was a consequence of the smaller initial mass of their pups relative to those of multiparous females (Table 3). Although primiparous grey seal females had a lower absolute daily milk energy output compared to multiparous females (Fig. 3A), there was no difference in mass-specific daily milk energy output (Fig. 3B) and, thus, relative pup growth or body composition (Table 3). These results suggest that the greater nursing effort of primiparous females compared to multiparous females [26] enables primiparous females and their pups to achieve the same relative rate of milk energy transfer as multiparous females. Although milk energy transfer to offspring was not directly measured, a higher nursing effort by primiparous females has also been noted in macaques (*Macaca sp.*, [45], [46]) suggesting that an increased nursing effort by primiparous females may also serve to compensate for a reduced physiological capacity for milk secretion in other taxa.

The strong relationship between maternal parturition mass and daily milk output in primiparous grey seal females (Fig. 4A) suggests that, consistent with observations for other species, the growth of mammary gland structural tissues through juvenile life and up to first conception are strongly correlated with increases in body size in grey seals (see [47]). As a result, grey seal females which are larger at first reproduction have relatively larger mammary glands and, thus, a greater daily milk output. A similar relationship between body weight at parturition and first lactation milk yield has also been noted for dairy cattle [48] suggesting that this relationship may be consistent among mammals. However, the lack of relationship between daily milk output and maternal parturition mass in multiparous grey seal females (Fig. 4A) suggests that, like laboratory species [15], [49], increases in the total number of secretory cells in the mammary gland with reproductive experience (until a maximum size is reached) will not be proportional to further increases in body size with age. Whether the magnitude of the increase in mammary gland size with subsequent cycles of pregnancy and lactation differs among individuals has not been investigated. Thus, whether a relatively high daily milk output at first lactation is predictive of a relatively high rate of output when the glands have reached their maximum size remains to be determined.

As a capital breeder (i.e. the nutrients used to support lactation are stored prior to parturition), the energy available to support both milk production and maternal maintenance in grey seals is limited by the total level of body energy stores at parturition. Behavioural observations suggested that, as a result of a higher level of activity during lactation, primiparous grey seal females may allocate a greater proportion of their initial energy stores to maternal maintenance costs versus milk production than multiparous females [26]. While the proportion of daily energy expenditure devoted to milk energy output between early and peak lactation averaged significantly less for primiparous females compared to multiparous females, there was a substantial overlap in the range between the groups and, overall, the efficiency of energy transfer to pups did not differ between primiparous and multiparous females (Table 4). This suggests that the difference in activity level between the groups is not sufficient to significantly influence overall lactation performance. In contrast to results for laboratory species in which primiparous females have a substantially lower transfer efficiency compared to multiparous females [39], [40], our results show that primiparous grey seal females do not need to expend energy at a proportionally higher rate to achieve the same relative rate of pup growth as multiparous females (Fig. 1B; Table 3). The reason for the lower transfer efficiency of primiparous females in laboratory species has not been investigated. It may reflect a reduced efficiency of energy acquisition and processing during lactation in non-capital breeders [50], [51], a failure to minimize the thermoregulatory losses of dependent offspring [52] and/or a substantially higher level of general activity due to increased sensitivity to disturbance [53].

In laboratory rats, primiparous females invested proportionally more energy than multiparous females to achieve the same level of offspring production [39]. Although laboratory species may exhibit trade-offs not seen in wild populations [54], [55], there is limited evidence that offspring production may also be more energetically costly for primiparous females in free-ranging golden mantled ground squirrels (*Spermophilis saturatus*; [56]). In the present study, the proportion of initial body energy stores mobilized to support the costs of lactation did not differ between primiparous and multiparous grey seals (Fig. 2). Thus, pup production was not more energetically costly for primiparous females. This contrast may reflect differences in life-history patterns. Small mammals have relatively high extrinsic mortality rates and, thus, primiparous females may invest relatively more in current reproduction because of a lower expectation of future reproduction [57]. In contrast, grey seals, like other large-bodied mammals, have low extrinsic mortality rates and a high expectation of future reproduction [8] and, thus, may not risk future survival or fecundity [58] by investing proportionally more energy than multiparous females in an effort to raise a larger pup with a greater probability of post-weaning survival. Alternatively, the level of energy investment over lactation may be physiologically limited in grey seals. Longitudinal data collected from individual grey seal females indicated that, although the proportion of body energy stores mobilized over lactation varied substantially among females, it was consistent within females across lactations [23]. These results suggested that the proportion of energy stores mobilized by individuals may act as a consistent signal for entrance into oestrus and the subsequent termination of lactation in fasting phocid seals [23]. Although further studies are needed, this suggests that, in contrast to species which offset the costs of lactation by increasing energy intake, there may be a limit on the proportion of energy stores expended by individual grey seal females.

Although the lactation length of fully-grown multiparous females was longer than that of primiparous females, the difference in average lactation length was not proportional to the difference in mass between the groups (Table 1, Fig. 4B). This is because the duration of lactation within individuals depends not only on the absolute level of body energy stores available at parturition but also on the proportion of initial body energy stores which are mobilized prior to weaning and on the rate at which those energy stores are used [23]. Daily milk energy output accounts for the greatest proportion of daily energy expenditure in grey seals [22]. Because of the smaller volume of secretory tissue in their mammary glands, the rate of milk production and, thus, daily milk energy output was significantly lower for primiparous females compared to multiparous females (Fig. 1A). Therefore, their absolute rate of daily energy expenditure was significantly less and the time it took to use the same proportion of body energy stores was greater than would be predicted by the difference in maternal postpartum mass alone (Fig. 4B). While the duration of lactation was approximately two days longer in multiparous females than in primiparous females, the majority of milk energy transfer was completed by day 12 postpartum with pups attaining approximately 90% of weaning mass in both groups. Consequently, the longer average lactation length of multiparous females was not sufficient to result in a significant difference in the relative mass or condition of pups at weaning (Table 3).

Although the body composition of pups of primiparous females did not differ from those of multiparous females, the post-weaning survival probability of the pups of primiparous females may none-the-less be lower than that of the pups of multiparous females (see [25], [59]). In phocid seals, the duration of the post-weaning fast plays a critical role in the development of a pup's oxygen storage capability which determines dive capacity and, in turn, will influence foraging ability. In grey seals, the duration of the post-weaning fast is correlated with percent body fat at weaning [24] which suggests that pups of primiparous females are capable of sustaining the post-weaning fast for a similar duration and, thus, are capable of developing the same relative oxygen storage capacity as those of multiparous females. However, because of their smaller body mass, the absolute oxygen storage capacity of the pups of primiparous females will be lower than that of the pups of multiparous females resulting in shorter or shallower dives and, therefore, less effective foraging. Nevertheless, given that mammary gland capacity increases with successive reproductive cycles, females which begin reproducing as soon as body size and energy reserves permit may gain an advantage over females which mature at a later age by developing their milk secretory capacity sooner, thereby increasing their subsequent maternal reproductive success and, potentially, lifetime fecundity.

Although the results of this study demonstrate that reproductive experience does not significantly influence the overall lactation performance of grey seals, they suggest that increases in mammary gland capacity with reproductive experience may play a significant role in the age-related increases in neonatal growth rates and weaning masses observed in other free-ranging mammals (e.g. [9], [10], [11]).

## Supporting Information

Figure S1 **Relationship between rate of mass gain and daily milk energy intake in grey seal pups.** Relationship between the rate of mass gain and daily milk energy intake between early and peak lactation in pups of primiparous (n = 14) and multiparous (n = 15) grey seal females (*y* = 0.027*x*+0.011).
(EPS)

Figure S2 **Relationship between mass at birth and subsequent daily milk intake in grey seal pups.** Relationship between mass at birth and the subsequent average daily milk intake of grey seal pups between (A) birth and 5 days of age (n = 18) and (B) between birth and 15 days of age (n = 17).
(EPS)

Text S1 **Relationship between mass at birth and the subsequent daily milk intake of grey seal (Halichoerus grypus) pups.**
(DOC)
