# Supplementary material for: The Influence of Reproductive Experience on Milk Energy Output and Lactation Performance in the Grey Seal (Halichoerus grypus)
Source: PLoS One. 2011 May 11;6(5):e19487. doi: 10.1371/journal.pone.0019487 (PMC3092752; doi:10.1371/journal.pone.0019487)
Supplement: Text S1 — Relationship between mass at birth and the subsequent daily milk intake of grey seal (Halichoerus grypus) pups. (DOC) [file pone.0019487.s003.doc]

**Supporting Information - Text S1.**

**Relationship between mass at birth and the subsequent daily milk intake of grey seal (*Halichoerus grypus*) pups.**

Studies in domestic species suggest that variation in the suckling demands of offspring can have a substantial impact on the rate of milk production with larger offspring associated with increased levels of daily milk output (e.g. [1]). However, the relationship between pup size at birth and the subsequent daily milk production of females has not been examined for any species of pinniped. Using longitudinal data collected by Mellish et al. ([2]) from 18 grey seal mother-pup pairs we examined whether pups which are larger at birth have a higher subsequent daily milk intake. We found no relationship between the mass of pups at birth and their subsequent average daily milk intake either during the early stages of lactation (between birth and 5 days of age; Fig. S2A) or over the course of lactation (between birth and 15 days of age; Fig. S2B). These results are consistent with the observation that birth mass and the subsequent growth rates of pups through lactation are not related in grey seals [3].

1. King RH, Mullan BP, Dunshea FR, Dove H (1997) The influence of piglet body weight on milk production of sows. Livestock Production Science 47: 169-174.

2. Mellish JE, Iverson SJ, Bowen WD (1999) Variation in milk production and lactation performance in grey seals and consequences for pup growth and weaning characteristics. Physiological and Biochemical Zoology 72: 677-690.

3. Smiseth PT, Lorensten S-H (1995) Evidence of equal maternal investement in the sexes in the polygynous and sexually dimorphic grey seal (*Halichoerus grypus*). Behavioral Ecology and Sociobiology 36: 145-150.
